# Case Report: Giant hydropic leiomyoma of the uterus presenting as an aggressive abdominopelvic tumor

**DOI:** 10.3389/fonc.2025.1529793

**Published:** 2025-10-22

**Authors:** Konstantinos Giannios, Elissavet Anestiadou, Eftychia Liampou, Lydia Konstantina Spilioti, Dimitra Rafailia Bakaloudi, Vasileios Papadopoulos

**Affiliations:** ^1^ 1st Department of Surgery, Aristotle University of Thessaloniki, Papageorgiou General Hospital, Thessaloniki, Greece; ^2^ 4th Academic Department of Surgery, Aristotle University of Thessaloniki, G.Papanikolaou General Hospital, Thessaloniki, Greece; ^3^ Département de Gynécologie-Obstétrique, Hôpital Bichat, Paris, France; ^4^ Division of Hematology and Oncology, Department of Medicine, University of Washington, Seattle, WA, United States

**Keywords:** uterine leiomyoma, hydropic degeneration, cystic degeneration, abdominopelvic mass, benign uterine tumors, hysterectomy, surgical resection

## Abstract

Hydropic leiomyoma (HLM) is a rare subtype of uterine leiomyoma characterized by significant interstitial fluid accumulation, often mimicking malignant tumors due to its imaging features. Although most uterine leiomyomas are benign and commonly occur in women of reproductive age, HLM can grow to an unusually large size, leading to diagnostic challenges. In this case report, we present a case of a 59-year-old postmenopausal woman with a giant HLM exhibiting extensive cystic hydropic degeneration resembling an aggressive abdominopelvic tumor. The tumor measured 35 × 27 × 17 cm and caused a significant mass effect on surrounding organs. Surgical management involved a total abdominal hysterectomy with right salpingo-oophorectomy via midline laparotomy. Intraoperative findings included displacement of the small bowel, transverse colon, and greater omentum by the tumor, with adherence of the left adnexa to the external surface of the uterus. A left ureteral transection occurred during tumor dissection and was successfully repaired with ureteral reanastomosis and placement of a pigtail stent. The operation lasted 4 hours 11 minutes, and the patient had an uncomplicated postoperative recovery. Histopathological examination confirmed the diagnosis of HLM with extensive cystic degeneration. Based on available literature, this case appears to represent the largest HLM reported to date, highlighting the importance of accurately distinguishing benign from malignant tumors to guide appropriate clinical management. This case underscores the complexities associated with diagnosing and surgically treating large, degenerating uterine leiomyomas.

## Introduction

Uterine leiomyomas, also known as fibroids or uterine myomas, are the most common benign neoplasm in women, occurring in 20%–30% of women, mainly in the 30–50 year age group (1). They are non-cancerous monoclonal tumors arising from smooth muscle cells and fibroblasts of the uterine wall (2).

In 80%–90% of cases, leiomyomas are of the conventional or usual type, with monotonous spindle cells, rare mitoses, and benign biological behavior. However, leiomyoma variants constitute a heterogeneous group with the same symptoms and signs as classic leiomyomas, but with malignant or uncertain potential (3). In addition, uterine leiomyomas can undergo different types of degeneration, including hyaline, cystic, hydropic, myxoid, fatty, hemorrhagic degeneration, or presentation of calcifications (4). These degenerative changes have been attributed to relative local ischemia during mass enlargement (5) and are of paramount importance in avoiding pitfalls in the differential diagnosis of uterine sarcoma and other ovarian tumors (6).

Uterine leiomyomas are classified by the International Federation of Gynaecology and Obstetrics (FIGO) into nine subtypes (0–8) based on their location within the uterus (7). These range from submucosal (types 0–2) and intramural (types 3–4) to subserosal (types 5–7) and those leiomyomas that do not relate to the myometrium at all, such as cervical, parasitic (type 8) (7). While hydropic leiomyomas (HLMs) are not explicitly categorized in this system, their large size and extensive degeneration often make them comparable to FIGO types 7 or 8, particularly when they grow exophytically or involve adjacent structures.

According to the 5th edition of the World Health Organization (WHO) classification of female genital tumors, HLM is a distinct, extremely rare leiomyoma subtype with significant interstitial fluid or stromal watery edema leading to the separation and compartmentalization of smooth muscle cells, increased vascularity, and arrangement of tumor cells in nodules or cords (8, 9). HLM tumors are significantly larger than usual-type leiomyomas, with a mean size of 14.4 cm (10). Presentation of imaging characteristics that can resemble malignancy renders differential diagnosis from malignant uterine tumors like leiomyosarcoma quite challenging. However, this distinction is critical for guiding treatment choices and ensuring appropriate patient follow-up. The literature contains a limited number of HLM cases, while reports of giant HLMs are extremely rare.

Hydropic degeneration refers to the accumulation of interstitial fluid and stromal edema within the uterine leiomyoma, leading to the expansion and softening of the tumor (10). While focal hydropic change is relatively common, certain rare and more extensive subtypes have been described, including diffuse hydropic degeneration and perinodular hydropic degeneration (PHD). Diffuse hydropic degeneration results in the widespread disruption of the classic smooth muscle architecture of the leiomyoma, often with prominent cystic spaces, vascular congestion, and soft, gelatinous consistency. PHDL is characterized by excessive interstitial fluid accumulation around smooth muscle bundles, creating a multinodular pattern that can mimic more aggressive or infiltrative tumors, such as intravenous leiomyomatosis or low-grade endometrial stromal sarcoma (8). These degenerative patterns are particularly important because they can significantly alter both the imaging characteristics and gross morphology of leiomyomas, complicating preoperative diagnosis and raising suspicion for malignancy. For this reason, early and precise recognition of such variants is essential in guiding appropriate surgical and pathological management.

Herein, we report an unusual case of a 59-year-old postmenopausal woman who presented with a giant uterine HLM with massive cystic hydropic degeneration mimicking an aggressive abdominopelvic tumor. The case underscores the diagnostic challenges, therapeutic considerations, and surgical complexities associated with the management of such massive tumors.

## Case description

A 59-year-old nulliparous woman presented to the outpatient clinic of our general surgery department complaining of an abdominopelvic mass that had been gradually increasing in size for the past 5 years. The patient also reported abdominal distension without any other symptoms. The patient had no history of chronic medical conditions, prior abdominal or pelvic surgeries, or hormone therapy. Her gynecologic history included regular menstrual cycles until menopause at age 49. She was nulliparous, with no history of infertility evaluation or assisted reproduction. There were no prior reports of abnormal uterine bleeding, pelvic pain, or known uterine pathology. She was a non-smoker, reported no alcohol or drug use, and had no family history of gynecologic or colorectal malignancies. Surgical history included an open appendectomy in childhood. Investigation of the abdominal mass began 1 month ago during hospital admission for renal colic of the right kidney.

The patient had first noticed gradual abdominal distension approximately 5 years ago but attributed it to aging, dietary habits, and minor weight gain. Because she remained largely asymptomatic and experienced no significant pain, bleeding, or gastrointestinal or urinary disturbances, she did not seek medical evaluation during that period. She had not undergone any imaging or gynecologic evaluation prior to the recent hospitalization. The presence of right-sided renal colic led to her first abdominal ultrasound, which incidentally revealed the large abdominopelvic mass and initiated further diagnostic investigation.

**Figure 1:**
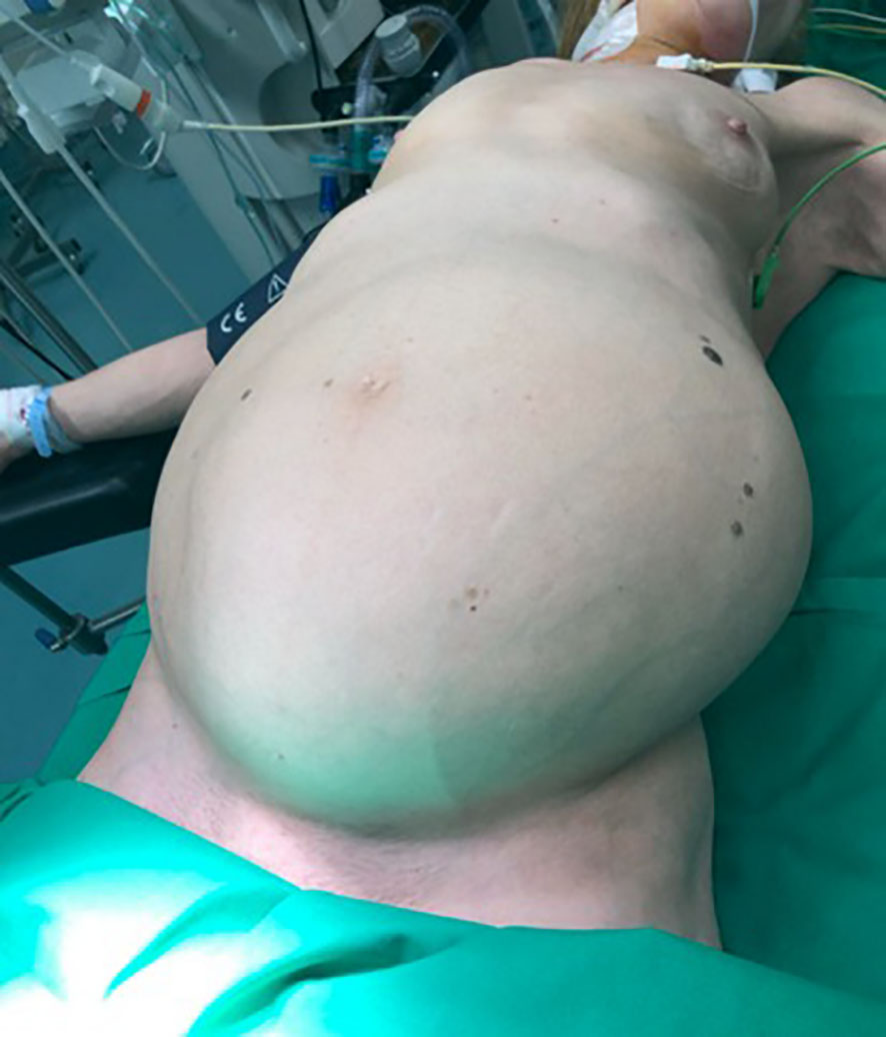
Significant distension of the abdomen, with overlying skin striae and dilated abdominal wall veins.

On physical examination, the patient was emaciated and frail with normal vital signs. Abdominal examination revealed significant abdominal distension due to a large, irregular, non-tender, and immobile lesion occupying the entire abdominal and pelvic cavity. Physical examination also revealed a dullness to percussion and a shifting dullness, indicating the presence of ascites. The overlying skin showed striae and dilated abdominal wall veins, consistent with abdominal distension and venous congestion (**Figure 1**). No abnormal lymph nodes were palpable.

Laboratory tests, including the tumor markers Cancer Antigen 125 (CA-125), Alpha-fetoprotein (AFP), Cancer Antigen 15-3 (CA15-3), and Cancer Antigen 19-9 (CA19-9), were within the normal range, whereas Carcinoembryonic Antigen (CEA) was slightly elevated (8.04 ng/mL).

The tumor markers were obtained as part of the preoperative evaluation to assess for potential gynecologic or gastrointestinal malignancy, given the presence of ascites and complex imaging features. While the CA-125, CA19-9, and AFP levels were within normal limits, CEA was mildly elevated. This finding was interpreted with caution, as low-level CEA elevation may occur in benign conditions involving chronic compression or inflammation of the bowel. In the absence of radiologic or clinical findings suggestive of gastrointestinal cancer, CEA was considered non-specific and not indicative of malignancy in this case.

**Figure 2:**
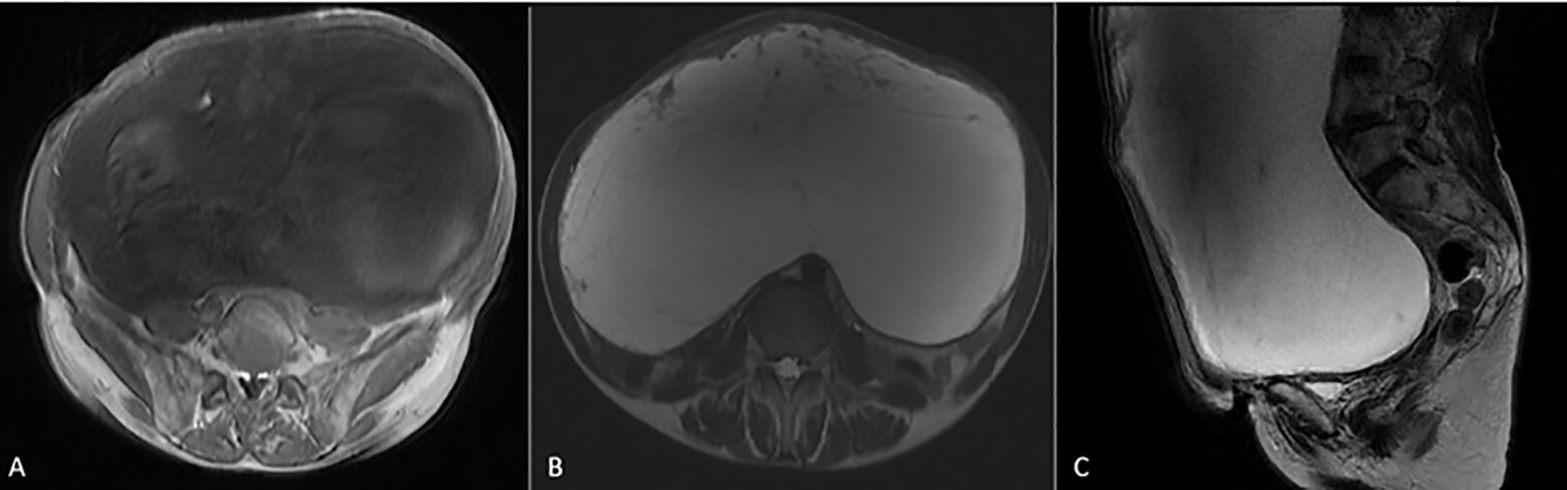
MRI images of the giant hydropic leiomyoma (HLM). **(A)** T1-weighted image showing the leiomyoma with low-to-intermediate signal intensity, reflecting the tumor’s solid components. **(B)** T2-weighted image demonstrating high signal intensity within the leiomyoma, indicative of significant fluid accumulation due to hydropic degeneration. **(C)** T2-weighted MRI in the sagittal plane showing a large HLM with high signal intensity. The mass causes significant mass effect on adjacent organs, displacing the bladder anteriorly and compressing the rectum posteriorly.

Abdominal ultrasound (US) showed a huge cystic mass occupying most of the abdominal cavity. Fatty liver infiltration with inhomogeneous parenchyma without dilatation of the intrahepatic biliary system, without cirrhosis, as well as microlithiasis of the kidneys with pelvicalyceal dilatation, especially on the right side, and abundant ascitic fluid in the peri-hepatic, hepato-renal, and peri-splenic spaces and in the pouch of Douglas were additional US findings. Computed tomography (CT) of the chest, abdomen, and pelvis showed a large complex cystic mass with calcifications occupying the entire abdomen and pelvis, causing compression of the adjacent abdominal organs. Intravenous contrast showed thick, enhancing septations. The ovaries and cervix were not visible due to displacement and compression. There was no evidence of intra-thoracic metastases or enlarged lymph nodes. Magnetic resonance imaging (MRI) of the abdomen and the pelvis revealed occupation of the greatest part of the abdomen and the pelvis by a huge abdominopelvic complex cystic mass, with a small number of solid parts and thick septations, as well as ascitic fluid in the pouch of Douglas. The mass measured approximately 27.7 cm in transverse diameter, 19 cm in anterolateral diameter, and 32 cm in cephalocaudal diameter (**Figures 2A–C**). Cytological examination was negative for malignancy, showing only rare mesothelial cells and sparse lymphocytes.

Despite the presence of imaging findings that raised suspicion for malignancy—such as the tumor’s large size, thick internal septations, and the presence of ascites—several characteristics supported a benign diagnosis. On MRI, the lesion demonstrated well-circumscribed margins without infiltrative behavior, and there were no signs of lymphadenopathy, necrosis, or peritoneal deposits. The solid components showed no irregular or intense enhancement, and the T2-weighted hyperintensity correlated with significant fluid accumulation rather than tumoral aggressiveness. These findings, when combined with normal CA-125 and the absence of systemic symptoms, favored a diagnosis of a benign but atypically degenerating uterine mass. Nonetheless, due to the size, mass effect, and residual diagnostic uncertainty, surgical resection was deemed necessary by the multidisciplinary team.

Based on preoperative imaging, the differential diagnosis included ovarian mucinous cystadenoma, uterine leiomyosarcoma, endometrial stromal sarcoma, and mesenteric cyst, and after multidisciplinary tumor board consultation and patient consent, surgical resection was decided. The case was reviewed by a multidisciplinary tumor board comprising a general surgeon, a gynecologic oncologist, a radiologist, and a pathologist. Imaging findings—including a large cystic mass with enhancing septations, absence of visible ovaries, and associated ascites—raised concern for malignancy, particularly ovarian epithelial tumors and uterine sarcomas. However, the absence of intrathoracic metastases, normal CA-125 levels, and a well-circumscribed border on MRI tempered suspicion for overt malignancy. Due to the tumor’s massive size, increasing abdominal discomfort, and diagnostic uncertainty, the consensus was to proceed with exploratory surgery and resection for both therapeutic and diagnostic purposes.

Under general anesthesia, the patient was placed in the supine position, and a midline laparotomy was performed via a midline incision. After entry into the peritoneal cavity, a giant cystic lesion was recognized, displaying the small bowel, the transverse colon, and the greater omentum to the left hypochondrium, while the body of the uterus and the right ovary were found in the right lateral abdomen. Investigation of the liver and the peritoneal cavity was negative for metastatic disease or lymphadenopathy. The left ovary was not recognized intraoperatively. Dissection of the cystic mass was performed in combination with total hysterectomy and right salpingo-oophorectomy, using a LigaSure™ vessel-sealing device (Medtronic, Covidien) for hemostasis and tissue sealing.

Both ureters were recognized intraoperatively, while the left ureter was transected during the dissection of the posterior surface of the tumor. The transection occurred despite prior identification of the ureter due to dense adhesions and anatomic distortion caused by the tumor’s size and posterior extension. The injury was immediately recognized and repaired intraoperatively with ureteral spatulation, reanastomosis using interrupted 4–0 PDS sutures, and placement of a 6-Fr pigtail stent. Preoperative prophylactic ureteral stenting was not performed, as the anatomy was presumed navigable on imaging; however, this approach may be reconsidered in similar future cases. Estimated blood loss was approximately 300 mL, and no blood transfusion was required. No other intraoperative complications occurred.

**Figure 3:**
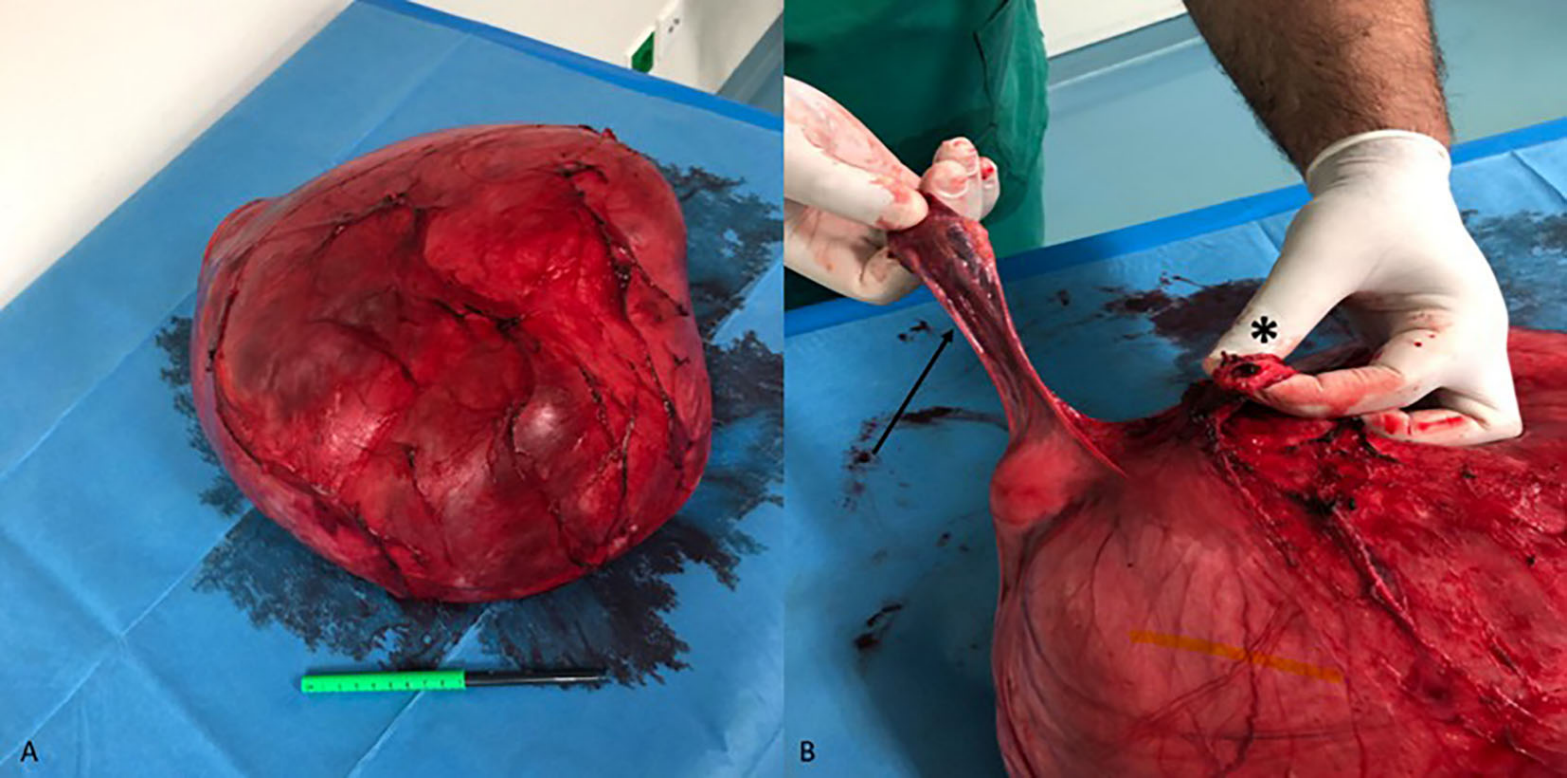
Uterine leiomyoma with diffuse hydropic degeneration. **(A)** Surgical specimen after total hysterectomy with right salpingo-oophorectomy. **(B)** The right adnexa (arrow) and the vaginal stump (asterisk) are recognizable on the specimen. Although the left ovary was not recognized intraoperatively, it was found to be completely adherent to the outer surface of the tumor on histological examination.

During anterior dissection, the tumor was successfully dissected off the bladder, and the cervix was identified. After ligation and division of the round, broad, sacro-uterine, and transverse cervical ligaments of the uterus, vaginal transection was performed. The specimen of the tumor *en bloc*, along with the uterus and the right ovary, was sent for histopathological examination (**Figures 3A, B**). The total operative time was 4 hours 11 minutes, and the patient was transferred to the ward without postoperative administration in the intensive care unit.

The mass measured 35 × 27 × 17 cm in diameter. On macroscopic examination, the specimen consisted of an encapsulated multilocular white tumor, predominantly cystic with gelatinous content and partly solid with fibrous-elastic structure. Its external surface was smooth and intact. The tumor was attached to the uterus and both adnexa. The left adnexa (ovary and fallopian tube) was completely adherent to the outer surface of the tumor. The tumor presented microscopic morphology suggestive of HLM with extensive cystic hydropic degeneration. Focal ischemic-type necroses were observed, while no cellular atypia or increased mitotic activity was noted. The cell proliferation index (Ki-67) reached up to 2%. Immunohistochemical stains were also indicative of HLM, with the following immunophenotype: desmin (+), smooth muscle actin (SMA) (+), calponin (+), Wilms tumor 1 (WT-1) (+), Estrogen Receptor (ER) +, Progesterone Receptor (PR) +), AE1/AE3 (−), inhibin (−), melan-A (−), and calretinin (−). The histological appearance of the ovaries and the fallopian tubes was age-consistent.

The patient had an uncomplicated postoperative course and was discharged on the fourth postoperative day, with no signs of complications during outpatient visits. The pigtail stent was removed following an ascending urethrocystography, which confirmed ureteral anastomotic integrity and the absence of leakage. A subsequent cystoscopy was then performed to complete the evaluation and facilitate the stent’s removal. On 6-month follow-up, she remained symptom-free, with no evidence of recurrence on ultrasound. A concise overview of the patient’s course is presented in **Table 1**.

**Table 1:** Abbreviated presentation of the patient’s course relative to day of surgery.

5 years prior	Gradual development of an abdominopelvic mass without other symptoms.
Day −30	Initial hospital admission for right-sided renal colic and initial evaluation of the abdominal mass.
Presentation	Abdominal distension with a large, irregular, immobile mass and signs of ascites.
Day −28	Abdominal ultrasound revealed large pelvic mass.
Day −26	CT scan performed; mass size and features noted.
Day −24	Tumor board discussion and decision for surgical management.
Day −22	MRI performed to further assess tumor characteristics.
Day −20	Preoperative plan: preoperative evaluation (laboratory tests, tumor markers, and anesthesia clearance). Differential diagnosis included ovarian or uterine malignancies; decision for surgical resection was made.
Day −1	Patient admitted for surgical preparation.
Day 0	Surgery: total abdominal hysterectomy with right salpingo-oophorectomy and left ureteral transection repair.
Postoperative Day 1	Stable recovery, no complications.
Postoperative Day 4	Discharged home in good general condition.
Postoperative Week 6	Outpatient follow-up, favorable recovery noted.
Postoperative Month 3	Follow-up imaging confirmed no recurrence or complications.

## Discussion

Uterine leiomyomas are the most common neoplasm in premenopausal women, with a lifetime incidence of approximately 70%, and they are more common in patients on progesterone therapy (11). Although the majority of cases are asymptomatic, approximately 30% of women with leiomyomas present with a range of serious symptoms, including abnormal uterine bleeding, anemia, pelvic or low back pain, urinary frequency and retention, constipation, obstetric complications, or infertility, particularly in the case of submucosal leiomyomas (12). Leiomyomas are the most common indication for hysterectomy in the United States, resulting in an annual economic burden of approximately $5.9–34.4 billion (USD) (13). To our knowledge, the present case represents the largest HLM reported in the literature, contributing to the limited body of literature on this rare clinical entity.

The classic (conventional or typical) spindled type of uterine leiomyoma is the most common form of leiomyoma, accounting for 80%–90% of leiomyomas (14). In contrast, leiomyoma variants are extremely rare, with an incidence of 1% to 2% (4). Most classic-type leiomyomas have a mitotic index of less than five mitotic figures per 10 high-power fields, mild cytological atypia, and no evidence of tumor cell necrosis (15). In contrast, leiomyoma variants are extremely rare, with an incidence of 1% to 2% (4). In addition, classic-type leiomyomas do not present a diagnostic dilemma on MRI and US imaging (16). On US, classic-type non-degenerated leiomyomas present as a round or oval, well-circumscribed, hypoechoic solid lesion, often accompanied by posterior shadowing due to the presence of calcifications or the interface of the leiomyoma-normal uterine wall (4). On MRI, a classical leiomyoma usually presents as a well-circumscribed mass with isointense or mildly low signal intensity (SI) on T1-weighted (T1W) images, characteristically low SI on T2-weighted (T2W) images, and enhancement after contrast administration (14). Histologically, leiomyomas are characterized by large amounts of extracellular matrix containing collagen, proteoglycan, and fibronectin and have a thin pseudocapsule containing areolar tissue and compressed muscle fibers (2).

Degenerative changes of leiomyomas are common, with an incidence of 10%, even within the same tumor, and are caused by the inadequate blood supply of leiomyomas, which are usually large or have ramified growth (6, 17). Degenerative changes result in a heterogeneous appearance on imaging, with minimal or irregular enhancement (18). Conventional-type leiomyomas may present a series of various degenerative changes. Hyaline is the most common type of degeneration (in 60% of cases) and is characterized by the presence of eosinophilic bands or plaques in the extracellular space (6, 19). Hemorrhagic (carneous or red) degeneration is common in pregnant women or women taking oral contraceptives and is caused by massive hemorrhagic infarction due to venous thrombosis in the periphery of the tumor (20). Myxoid degeneration is extremely rare and is characterized by smooth muscle cells with a significant concentration of cell-rich acid mucin (21). Fatty degeneration or lipoleiomyoma is a rare type of tumor caused by fatty metamorphogenesis of smooth muscle cells and is composed of adipocytes and smooth muscle cells separated by thin fibrous septa (22). Finally, hydropic degeneration results from extensive fluid accumulation and watery edema within the leiomyoma (23).

Focal hydropic degeneration is commonly encountered, occurring in up to 50% of fibroids (23). However, diffuse hydropic cystic degeneration and PHDL are also two rare presentation subtypes of hydropic degeneration (6). More specifically, diffuse hydropic cystic degeneration leads to extensive obliteration of the common fibroid architecture, with the presence of numerous thick-walled blood vessels distorting its smooth muscle origin (8). PHDL is caused by increased fluid accumulation around the fascicles of the smooth muscle bundles, resulting in a multinodular growth pattern. The microscopic and macroscopic features of PHDLs pose a significant challenge in their differential diagnosis with intravenous leiomyomatosis, endometrial stromal sarcoma, or myxoid leiomyosarcoma (24). The aforementioned rare types of hydropic degeneration can lead to an excessive increase in tumor size due to massive intra-tumoral watery edema, with consequent diagnostic difficulties regarding their origin and biological behavior, similar to our patient (25). The extensive accumulation of interstitial fluid, as well as the cystic degeneration, can alter classic leiomyoma features, making differential diagnosis difficult. The risk of misdiagnosis is particularly high when evaluating tumor size, vascularity, and cellular morphology. In such cases, immunohistochemical analysis holds a crucial role in distinguishing HLM from other malignant lesions. Markers such as desmin, SMA, and calponin confirm smooth muscle origin, while a low Ki-67 proliferation index (<5%) supports a benign diagnosis (26). In this case, a broad immunohistochemical panel was applied not only to confirm smooth muscle differentiation but also to exclude other potential differential diagnoses given the tumor’s size, cystic appearance, and the patient’s postmenopausal status. AE1/AE3 was used to exclude an epithelial neoplasm, while inhibin and melan-A were employed to rule out sex cord stromal tumors, such as granulosa cell tumors. Calretinin and WT-1, although sometimes positive in smooth muscle tumors, were also useful in excluding a mesothelial origin or serous carcinoma, particularly given the presence of ascites and adnexal adhesions. This comprehensive panel supports the pathological conclusion of a benign hydropic leiomyoma, despite its unusual gross and radiologic features. The use of Ki-67 as a proliferation marker was of particular diagnostic value. According to existing histopathological criteria, leiomyomas typically exhibit a Ki-67 index of <5%, whereas higher indices (>10%–15%) are suggestive of malignant smooth muscle tumors such as leiomyosarcoma or smooth muscle tumors of uncertain malignant potential (STUMP) (15, 27). In our case, the Ki-67 index was 2%, consistent with low proliferative activity and reinforcing the benign diagnosis. The absence of cytological atypia, tumor cell necrosis, and increased mitotic activity further supported this interpretation. Thus, Ki-67 served as a practical and clinically meaningful tool to distinguish this degenerating leiomyoma from its malignant mimics. Consultation of a specialized pathologist with expertise in gynecologic pathology is advisable in cases with ambiguous histological features to ensure diagnostic accuracy and avoid unnecessary overtreatment.

The imaging and histologic findings in this case show a clear radiologic–pathological correlation. On MRI, the lesion demonstrated marked T2-weighted hyperintensity (**Figures 2B, C**), which corresponds to the extensive stromal edema and cystic fluid accumulation seen histologically—hallmark features of hydropic degeneration. Additionally, the compartmentalized, nodular architecture identified on microscopic examination explains the septated and multilocular appearance of the tumor on imaging. Grossly, the tumor’s smooth, lobulated external surface (**Figure 3A**) supports its benign, encapsulated nature. Immunohistochemical staining further confirmed a smooth muscle origin (desmin+, SMA+, and calponin+) with low proliferative activity (Ki-67 < 5%), consistent with a diagnosis of benign hydropic leiomyoma and excluding high-grade malignancy.

HLMs may present on MRI with imaging features that overlap with malignant tumors, particularly leiomyosarcoma, endometrial stromal sarcoma, and ovarian cystic malignancies. Common features that raise suspicion for malignancy include large size, thick septations, cystic-solid architecture, and associated ascites. In our case, these characteristics necessitated careful preoperative evaluation and tumor board discussion (11). However, several radiologic findings were more suggestive of a benign process. These included the well-defined tumor margins, lack of infiltrative behavior, absence of intratumoral necrosis, and no evidence of pelvic or para-aortic lymphadenopathy or peritoneal nodules. Additionally, there was no enhancement of solid components in a pattern typical of aggressive sarcomas. The T2-weighted hyperintensity corresponded histologically to interstitial fluid accumulation and stromal edema, which are hallmark features of hydropic degeneration. Together, these imaging characteristics—although atypical—favored the diagnosis of a benign but degenerative leiomyoma, supporting the decision to proceed with surgical management. To further assist in differential diagnosis, a comparative summary of radiologic and clinical features distinguishing HLM from other cystic abdominopelvic masses is presented in **Table 2**.

**Table 2:** Differential diagnostic features of hydropic leiomyoma and other cystic abdominopelvic tumors.

Feature	Hydropic leiomyoma (HLM)	Uterine leiomyosarcoma	Ovarian cystadenocarcinoma	Mesenteric cyst
Patient demographics	Women, typically 30–60 years	Women >40 years	Women >40 years	All ages (rare)
Growth pattern	Slow, progressive; often very large	Rapid, aggressive	Variable; can be rapid	Usually slow
Margins on imaging	Well-defined, encapsulated	Poorly defined, infiltrative	Irregular, papillary projections	Well-circumscribed
MRI signal (T2)	High SI (due to edema/cystic change)	Heterogeneous; necrosis/hemorrhage	High SI with solid enhancing areas	Homogeneous high SI
Enhancement	Septal or rim enhancement; mild	Irregular, heterogeneous solid enhancement	Solid areas with strong enhancement	Thin rim, no solid enhancement
Ascites	May be present (hydropic pressure)	Often present (malignant effusion)	Often present	Rare
Lymphadenopathy	Absent	Common	May be present	Absent
Tumor markers	CA-125 usually normal; CEA may be mildly elevated	Non-specific; LDH may be ↑	CA-125, CEA often ↑	Negative
Histology	Smooth muscle cells with stromal edema and low mitotic index	Atypia, necrosis, high mitotic index	Epithelial origin, cytological atypia	Benign epithelial or lymphatic lining
Immunohistochemistry	Desmin+, SMA+, low Ki-67 (<5%)	Ki-67 often >10%, p53+, variable SMA	CK7+, CA-125+, WT-1+	Variable, non-specific

HLM, hydropic leiomyoma; SI, signal intensity; SMA, smooth muscle actin; CEA, carcinoembryonic antigen; CK7, cytokeratin 7; WT-1, Wilms tumor 1 protein; LDH, lactate dehydrogenase.

Based on its clinical and radiologic features, the HLM in this case aligns most closely with a FIGO type 7 or 8 leiomyoma, given its predominantly subserosal and exophytic growth pattern, with significant distortion of the uterus and involvement of adjacent structures (7). The extensive hydropic degeneration further complicates classification, as it contributes to the tumor’s massive size and fluid accumulation, features that are not explicitly addressed in the current FIGO system. This case underscores the need for enhanced classification criteria for rare leiomyoma variants such as HLM, particularly those presenting with aggressive mass effects and atypical degenerative changes. Although the FIGO classification system is a valuable tool for categorizing uterine leiomyomas based on their anatomic location, it does not account for critical characteristics such as tumor volume, extent of degeneration, or displacement into extrauterine spaces, all of which are highly relevant in cases of giant or hydropically degenerating leiomyomas (7). In our case, the tumor demonstrated extensive exophytic growth, stromal edema, and cystic changes, which are not explicitly addressed within the current FIGO types (0–8). We suggest that future iterations of the FIGO system could incorporate modifiers or subclassifications to reflect tumor size, degeneration type (e.g., hydropic, cystic, and myxoid), and anatomic distortion, as these factors have important implications for surgical strategy, differential diagnosis, and reporting consistency across studies.

The clinical presentation of HLMs is similar to that of classical leiomyomas, as reported by Clement et al. in the first case series of 10 patients (8). However, there are reports in the literature of HLMs presenting as pseudo-Meigs syndrome with dyspnea, pleural effusion, and ascites, which is a diagnostic pitfall in the differential diagnosis with other uterine and ovarian malignancies (28, 29). In addition, several cases of pregnant women with HLMs have been described (30, 31). Lai et al. described a case of retroperitoneal HLM preoperatively diagnosed as an ovarian cyst in a 46-year-old woman (32).

When evaluating large abdominopelvic masses, the differential diagnosis should extend beyond malignant conditions such as leiomyosarcoma or ovarian neoplasms to include also some benign but potentially complex presentations of large uterine leiomyomas, which may mimic more aggressive pathology. Among the latter, leiomyoma torsion is a rare but severe complication, occurring when a pedunculated subserosal fibroid twists around its vascular stalk, leading to impaired blood flow and necrosis (33). Clinical symptomatology often includes severe abdominopelvic pain, and imaging findings are suggestive of compromised blood flow, although definitive diagnosis is frequently made intraoperatively (33). Furthermore, leiomyoma torsion has been described in patients with syndromic conditions, such as in Mayer–Rokitansky–Küster–Hauser (MRKH) syndrome. In such cases, leiomyomas may arise in rudimentary uterine structures and undergo torsion, leading to acute abdominal pain and requiring prompt surgical intervention (34). Finally, large degenerative cystic leiomyomas often mimic ovarian neoplasms due to their mixed solid and cystic components. These lesions may cause significant mass effect to adjacent organs, leading to symptoms such as dyspnea, abdominal distension, and pressure-related organ dysfunction, which can complicate preoperative differential diagnosis from malignancy (35). Given the potential overlap in clinical presentation among these conditions, a thorough diagnostic approach incorporating detailed imaging, intraoperative assessment, and histopathological confirmation is essential for accurate differentiation.

HLMs are generally characterized by a larger size compared to classic-type leiomyomas. According to Griffin et al., HLMs have a mean size of 14.4 ± 8.2 cm, compared to a mean size of 6.7 ± 0.8 cm for classic-type leiomyomas (10). The literature reports limited data on giant HLMs. The first relative case report dates back to 1994, when Moore et al. reported a giant uterine leiomyoma with focal hydropic degeneration in a young pregnant woman (31). Horta et al. described a rare case of a 35-year-old patient with a giant pedunculated uterine leiomyoma with diffuse hydropic degeneration measuring approximately 20 × 30 × 8 cm who was treated with fertility-sparing myomectomy (36). Akkour et al. in 2021 reported a case of a 32-year-old woman with large pelvoabdominal masses measuring 33 × 24 × 15 cm in total who was treated with myomectomy with transposition of the ovaries to the lateral abdominal wall. Histopathology revealed a uterine leiomyoma with massive cystic hydropic degeneration (6). In a case report by Ye et al., a degenerated leiomyoma with extensive edema measuring 30 × 25 × 16 cm causing uterine torsion was resected in a postmenopausal woman who was treated with total abdominal hysterectomy (37). Based on published reports available in the literature, the present case appears to represent one of the largest HLMs documented to date.

As of today, there is no consensus on the optimal management of HLMs, which mainly includes the options of myomectomy and total abdominal hysterectomy with or without bilateral salpingo-oophorectomy (10). The decision between myomectomy and hysterectomy is influenced by various parameters such as patient age, tumor size and macroscopic appearance, willingness for fertility preservation, and intraoperative findings. In younger patients with fertility concerns, myomectomy should be preferred, provided that the tumor is well-demarcated and malignancy is not suspected. On the contrary, in postmenopausal women or cases of enlarged tumors with extensive adhesions rendering uterine preservation challenging, hysterectomy is proposed to ensure complete, margin-negative tumor resection and to reduce the risk of recurrence. In our case, a total abdominal hysterectomy was chosen due to the enlarged abdominopelvic tumor, its extensive cystic degeneration, and its mass effect on adjacent organs. Given the patient’s postmenopausal status and the increased risk of future adnexal pathology, right salpingo-oophorectomy was performed to facilitate complete tumor excision and optimize long-term outcomes. Postoperative course of HLMs was uncomplicated in the case series of Clement et al. and in a few case reports reported in the literature (8, 28, 30–32, 36, 37). Surgical management of uterine fibroids must be tailored to tumor size and complexity, particularly in rare cases like hydropic leiomyoma. Recent reviews highlight the evolving role of both open and minimally invasive approaches in fibroid treatment (38). The growing spectrum of available medical and surgical options for uterine fibroids underscores the importance of individualized treatment planning. Even in rare variants like hydropic leiomyoma, awareness of evolving therapeutic strategies can aid in selecting the most appropriate and safe approach (39).

This case report provides valuable insights into the rare presentation of giant HLM, contributing to the limited literature on this uncommon subtype of uterine leiomyoma. Based on available reports in the literature, this case appears to represent one of the largest HLMs documented to date. It contributes meaningfully to the limited body of knowledge on this rare clinical entity and serves as a reminder that even benign tumors may present with aggressive features, necessitating individualized surgical planning and multidisciplinary management. Surgeons managing large abdominopelvic masses should maintain a broad differential diagnosis and be prepared for unexpected findings, ensuring optimal patient outcomes through precise surgical execution and postoperative care.

A major strength is the detailed clinical and surgical documentation, which emphasizes the diagnostic challenges and surgical considerations required for such large, hydropic tumors, especially when they mimic malignancies. The report also highlights the successful management of intraoperative complications, underscoring the importance of multidisciplinary planning and expertise in managing complex cases. In this case, a total abdominal hysterectomy with right salpingo-oophorectomy was performed, along with ureteral reconstruction following iatrogenic injury, highlighting the complexities inherent to these extensive surgical procedures.

The operative management of giant HLMs poses significant technical challenges. Distorted anatomy, particularly the displacement of ureters and adnexa, increases the risk of intraoperative complications. In our case, an iatrogenic ureteral transection occurred despite careful dissection, highlighting the need for heightened vigilance in such anatomically complex settings. Although preoperative ureteral stenting was not performed, it may be advisable in similar cases with anticipated pelvic distortion. Intraoperative bleeding was moderate, and no transfusion was needed; however, surgeons must be prepared for substantial hemorrhage and potential visceral injury during resection of such massive tumors.

The patient’s successful postoperative recovery reinforces the value of a multidisciplinary approach and careful intraoperative techniques. However, a limitation is that the report represents a single case, limiting the generalizability of findings. Further research is needed in this field to better understand the pathophysiology and optimal management strategies for HLMs, particularly those of massive size.

In conclusion, HLMs, particularly those of considerable size, pose unique diagnostic and therapeutic challenges for surgeons. The potential for these tumors to mimic malignant processes underscores the critical importance of preoperative imaging and thorough histopathological assessment in order to guide surgical decision-making. In cases of giant HLMs, as presented in this report, the significant mass effect on surrounding organs requires meticulous preoperative planning and a highly skilled surgical approach to ensure complete resection while minimizing complications.

## Data Availability

The original contributions presented in the study are included in the article/supplementary material. Further inquiries can be directed to the corresponding author.

## References

[1] Li B Wang F Chen L Tong H. Global epidemiological characteristics of uterine fibroids. Arch Med Sci. (2023) 19:1802–10. doi: 10.5114/aoms/171786, PMID: 38058724 PMC10696973

[2] Holdsworth-Carson SJ Zaitseva M Vollenhoven BJ Rogers PAW. Clonality of smooth muscle and fibroblast cell populations isolated from human fibroid and myometrial tissues. Mol Hum Reprod. (2014) 20:250–9. doi: 10.1093/molehr/gat083, PMID: 24243625

[3] Alran L Rychlik Agnieszka CS. Leiomyoma-General . Available online at: https://www.pathologyoutlines.com/topic/uterusleiomyoma.html (Accessed May 13, 2025).

[4] Arleo EK Schwartz PE Hui P McCarthy S. Review of leiomyoma variants. Am J Roentgenol. (2015) 205:912–21. doi: 10.2214/AJR.14.13946, PMID: 26397344

[5] Dancz CE Macdonald HR. Massive cystic degeneration of a pedunculated leiomyoma. Fertil Steril. (2008) 90:1180–1. doi: 10.1016/j.fertnstert.2007.12.046, PMID: 18279856

[6] Akkour K Alhulwah M Alqahtani N Arafah MAA. Giant leiomyoma with massive cystic hydropic degeneration mimicking an aggressive neoplasm: A challenging case with a literature review. Am J Case Rep. (2021) 22:1–5. doi: 10.12659/AJCR.929085, PMID: 33785706 PMC8019839

[7] Gomez E Nguyen M-LT Fursevich D Macura K Gupta A. MRI-based pictorial review of the FIGO classification system for uterine fibroids. Abdom Radiol (N Y). (2021) 46:2146–55. doi: 10.1007/s00261-020-02882-z, PMID: 33385249

[8] Clement PB Young RH Scully RE. Diffuse, perinodular, and other patterns of hydropic degeneration within and adjacent to uterine leiomyomas. Problems in differential diagnosis. Am J Surg Pathol. (1992) 16:26–32. doi: 10.1097/00000478-199201000-00004, PMID: 1309411

[9] Höhn AK Brambs CE Hiller GGR May D Schmoeckel E Horn L-C. 2020 WHO classification of female genital tumors. Geburtshilfe Frauenheilkd. (2021) 81:1145–53. doi: 10.1055/a-1545-4279, PMID: 34629493 PMC8494521

[10] Griffin BB Ban Y Lu X Wei J-J. Hydropic leiomyoma: A distinct variant of leiomyoma closely related to HMGA2 overexpression. Hum Pathol. (2019) 84:164–72. doi: 10.1016/j.humpath.2018.09.012, PMID: 30292626 PMC6408288

[11] Lameira P Filipe J Cabeçadas J Cunha TM. Hydropic leiomyoma: A radiologic pathologic correlation of a rare uterine tumor. Radiol Case Rep. (2022) 17:3151–6. doi: 10.1016/j.radcr.2022.06.008, PMID: 35801124 PMC9253554

[12] Giuliani E As-Sanie S Marsh EE. Epidemiology and management of uterine fibroids. Int J Gynecol Obstet. (2020) 149:3–9. doi: 10.1002/ijgo.13102, PMID: 31960950

[13] Styer AK Rueda BR. The epidemiology and genetics of uterine leiomyoma. Best Pract Res Clin Obstet Gynaecol. (2016) 34:3–12. doi: 10.1016/j.bpobgyn.2015.11.018, PMID: 26725703

[14] Tu W Yano M Schieda N Krishna S Chen L Gottumukkala RV. Smooth muscle tumors of the uterus at MRI: focus on leiomyomas and FIGO classification. Radiographics. (2023) 43. doi: 10.1148/rg.220161, PMID: 37261965

[15] Bell SW Kempson RL Hendrickson MR. Problematic uterine smooth muscle neoplasms. A clinicopathologic study of 213 cases. Am J Surg Pathol. (1994) 18:535–58. doi: 10.1097/00000478-199406000-00001, PMID: 8179071

[16] Murase E Siegelman ES Outwater EK Perez-Jaffe LA Tureck RW. Uterine leiomyomas: histopathologic features, MR imaging findings, differential diagnosis, and treatment. RadioGraphics. (1999) 19:1179–97. doi: 10.1148/radiographics.19.5.g99se131179, PMID: 10489175

[17] Bura V Pintican RM David RE Addley HC Smith J Jimenez-Linan M. MRI findings in-between leiomyoma and leiomyosarcoma: A rad-path correlation of degenerated leiomyomas and variants. Br J Radiol. (2021) 94:20210283. doi: 10.1259/bjr.20210283, PMID: 34289327 PMC9327769

[18] Han SC Kim M-D Jung DC Lee M Lee MS Park S. Degeneration of leiomyoma in patients referred for uterine fibroid embolization: incidence, imaging features and clinical characteristics. Yonsei Med J. (2013) 54:215–9. doi: 10.3349/ymj.2013.54.1.215, PMID: 23225822 PMC3521269

[19] Ueda H Togashi K Konishi I Kataoka ML Koyama T Fujiwara T. Unusual appearances of uterine leiomyomas: MR imaging findings and their histopathologic backgrounds. Radiogr Rev Publ Radiol Soc North Am Inc. (1999) 19:S131–45. doi: 10.1148/radiographics.19.suppl_1.g99oc04s131, PMID: 10517450

[20] Takeuchi M Matsuzaki K Bando Y Harada M. Evaluation of red degeneration of uterine leiomyoma with susceptibility-weighted MR imaging. Magn Reson Med Sci. (2019) 18:158–62. doi: 10.2463/mrms.mp.2018-0074, PMID: 30270253 PMC6460131

[21] Panda A Mahoorkar DN Reddy N Reddy BM. Myxoid degeneration of leiomyoma-a masquerader. Int J Reprod Contraception Obstet Gynecol. (2022) 11:3415. doi: 10.18203/2320-1770.ijrcog20223145

[22] Nazir HM Mehta S Seena CR Kulasekaran N. Uterine Lipoleiomyoma: A report of two cases. J Clin Imaging Sci. (2017) 7:26. doi: 10.4103/jcis.JCIS_13_17, PMID: 28717557 PMC5508403

[23] Patil AR Nandikoor S Padilu R. Hydropic degeneration of leiomyoma in nongravid uterus: the “Split fiber” Sign on magnetic resonance imaging. Indian J Radiol Imaging. (2018) 28:182–6. doi: 10.4103/ijri.IJRI_472_17, PMID: 30050241 PMC6038210

[24] Kim S-N Jang J Kim K-R. Uterine leiomyomas with perinodular hydropic degeneration: A report of two cases. Korean J Pathol. (2002) 36:257–61.

[25] El Ghali EH Meziyane J Auragh S Saadi H Taheri H Mimouni A. Hydropic leiomyoma, a considerable differential diagnosis: A case series. Int J Reprod Contraception Obstet Gynecol. (2022) 11:1767. doi: 10.18203/2320-1770.ijrcog20221455

[26] Miettinen M. Immunohistochemistry of soft tissue tumours - review with emphasis on 10 markers. Histopathology. (2014) 64:101–18. doi: 10.1111/his.12298, PMID: 24111893 PMC7670586

[27] Oliva E. Practical issues in uterine pathology from banal to bewildering: the remarkable spectrum of smooth muscle neoplasia. Mod Pathol. (2016) 29:S104–20. doi: 10.1038/modpathol.2015.139, PMID: 26715170

[28] Zou L Lou J Huang H Xu L. Pseudo-meigs syndrome caused by a rapidly enlarging hydropic leiomyoma with elevated CA125 levels mimicking ovarian Malignancy: A case report and literature review. BMC Womens Health. (2024) 24:445. doi: 10.1186/s12905-024-03285-8, PMID: 39112955 PMC11304927

[29] Pauls M MacKenzie H Ramjeesingh R. Hydropic leiomyoma presenting as a rare condition of pseudo-meigs syndrome: literature review and a case of a pseudo-meigs syndrome mimicking ovarian carcinoma with elevated CA125. BMJ Case Rep. (2019) 12. doi: 10.1136/bcr-2018-226454, PMID: 30635302 PMC6340602

[30] Heffernan E Köbel M Spielmann A. Case report: hydropic leiomyoma of the uterus presenting in pregnancy: imaging features. Br J Radiol. (2009) 82:e164–7. doi: 10.1259/bjr/50866065, PMID: 19592400

[31] Moore L Wilson S Rosen B. Giant hydropic uterine leiomyoma in pregnancy: unusual sonographic and doppler appearance. J Ultrasound Med. (1994) 13:416–8. doi: 10.7863/jum.1994.13.5.416, PMID: 8015054

[32] Lai PH Ding DC. Retroperitoneal hydropic leiomyoma mimicking an ovarian cyst. Case Rep Obstet Gynecol. (2022) 2022:1–4. doi: 10.1155/2022/2012376, PMID: 35966885 PMC9371877

[33] Khaoula M Slama F Dhaoui S Khayati W Sana M Abir K. Uterine leiomyoma torsion: A rare cause of acute abdominal pain. Int J Surg Case Rep. (2024) 122:109788. doi: 10.1016/j.ijscr.2024.109788, PMID: 39032352 PMC11315058

[34] Romano F Carlucci S Stabile G Mirenda G Mirandola M Mangino FP. The rare, unexpected condition of a twisted leiomyoma in mayer-rokitansky-küster-hauser (MRKH) syndrome: etiopathogenesis, diagnosis and management. Our experience and narrative review of the literature. Int J Environ Res Public Health. (2021) 18. doi: 10.3390/ijerph18115895, PMID: 34072739 PMC8198036

[35] Hoang VT Hoang TH Van HAT Pham NTT Chansomphou V Nguyen TTT. Giant degenerative uterine leiomyoma mimicking an ovarian neoplasm: case report. SAGE Open Med Case Rep. (2025) 13:2050313X251315066. doi: 10.1177/2050313X251315066, PMID: 39866281 PMC11758534

[36] Horta M Cunha TM Oliveira R Magro P. Hydropic leiomyoma of the uterus presenting as a giant abdominal mass. BMJ Case Rep. (2015) 2015:1–5. doi: 10.1136/bcr-2015-211929, PMID: 26351316 PMC4567748

[37] Ye Z Jiang Y Yan K Yu C. Uterine torsion with degeneration and infarction of giant leiomyoma in a postmenopausal woman: A case report. Medicine (Baltimore). (2023) 102:e35964. doi: 10.1097/MD.0000000000035964, PMID: 37960802 PMC10637470

[38] Cianci S Gulino FA Palmara V La Verde M Ronsini C Romeo P. Exploring surgical strategies for uterine fibroid treatment: A comprehensive review of literature on open and minimally invasive approaches. (2024) 60(1):64. doi: 10.3390/medicina60010064, PMID: 38256325 PMC10820219

[39] Mici J Arsenijevi V Šljivan U. Currently available treatment modalities for uterine fibroids. (2024), 60(6):868. doi: 10.3390/medicina60060868, PMID: 38929485 PMC11205795

